# Different Responses of Left Atrium and Left Atrial Appendage to Radiofrequency Catheter Ablation of Atrial Fibrillation: a Follow Up MRI study

**DOI:** 10.1038/s41598-018-26212-y

**Published:** 2018-05-18

**Authors:** Yun Gi Kim, Jaemin Shim, Suk-Kyu Oh, Hee-Soon Park, Kwang-No Lee, Sung Ho Hwang, Jong-Il Choi, Young-Hoon Kim

**Affiliations:** 1 Division of Cardiology, Department of Internal Medicine, Korea University Medical Center Anam Hospital, Seoul, Republic of Korea; 2 Department of Radiology, Korea University Medical Center Anam Hospital, Seoul, Republic of Korea

## Abstract

Atrial fibrillation (AF) is known to cause adverse remodeling of left atrium (LA). Radiofrequency catheter ablation (RFCA) of AF is associated with decrease in LA volume. However, the impact of RFCA on left atrial appendage (LAA) volume and hemodynamic function is not fully understood. We analyzed 123 patients who underwent cardiac magnetic resonance imaging (MRI) evaluation before and after RFCA in Korea University Anam Hospital. LA and LAA volume were measured before and after RFCA based on cardiac MRI. Baseline LA volume was 99.5 ± 38.4 cm^3^ and decreased to 74.6 ± 28.5 cm^3^ after RFCA (p < 0.001). LA diameter measured with transthoracic echocardiography was also decreased after RFCA (43.3 ± 6.2 mm at baseline and 39.9 ± 5.9 mm at follow up; p < 0.001). However, LAA volume was significantly increased after RFCA (19.4 ± 8.5 cm^3^ at baseline and 23.7 ± 13.3 cm^3^ at follow up; p < 0.001). Total ablation time and additional substrate modification was associated with change in LA volume. After RFCA, average LAA velocity measured by transesophageal echocardiography was increased to 51.0 cm/sec from 41.1 cm/sec (p < 0.001). In conclusion, LAA volume was increased after RFCA in contrast to LA volume. Our data raise a concern about worsening hemodynamics of LA and LAA following RFCA and long term clinical significance of enlarged LAA after RFCA needs further evaluation.

## Introduction

Radiofrequency catheter ablation (RFCA) has emerged as a treatment of choice for antiarrhythmic drug (AAD) refractory symptomatic atrial fibrillation (AF) patients^1^. It is evident that RFCA is superior as compared with AAD for restoration and maintenance of sinus rhythm in AF patients^2,3^. Furthermore, RFCA is associated with significant improvements in quality of life^1,4^ and recent retrospective studies suggest that RFCA might also reduce ischemic stroke and all-cause mortality^5–8^.

Studies based on echocardiography and autopsy revealed that left atrial appendage (LAA) is the origin of thrombus formation in more than 90% of patients with non-valvular AF^9–12^. Thrombus and spontaneous echocontrast (SEC) in LAA and reduced LAA flow velocity are all associated with ischemic stroke in AF patients^13–15^. According to the SPAF-III study, annual risk of ischemic stroke in patients with dense SEC might reach 18.2% if adequate anticoagulation treatment is not given^16^. Additionally, LAA flow velocity less than 20 cm/sec was associated with 2.6 times increased risk of ischemic stroke^16^. Therefore, flow stasis in LAA and its consequences, which is SEC and thrombus formation, are important risk factors for developing clinical ischemic stroke. Recent studies suggested that LAA orifice area and LAA volume are also associated with increased risk of ischemic stroke^17,18^. Previous studies suggest that RFCA is associated with reverse remodeling of left atrium (LA)^19–24^. However, recent study performed by Wylie *et al*.^25^ showed that RFCA is associated with reduced atrial systolic function despite decreased LA volume which suggests that LA scar formation and consequent stiff LA are the principal mechanism for decreased LA volume following RFCA. Impaired LA function might predispose LAA to higher intraluminal pressure and may cause LAA to dilate similar to a balloon effect. Currently, the impact of RFCA on LAA is not fully revealed. Understanding the hemodynamic and mechanical impact of RFCA on LAA would be important to anticipate future risk of ischemic stroke and to determine anticoagulation strategy in AF patients. We performed this study to evaluate the influence of RFCA on both LA and LAA in AF patients.

## Methods

### Patients

Patients with AAD resistant AF undergoing first-time RFCA in Korea University Medical Center Anam Hospital were screened for their eligibility. All patients who underwent both baseline (pre-RFCA) and follow up (post-RFCA) cardiac magnetic resonance imaging (MRI) evaluation suitable for LAA size evaluation were enrolled. There was no specific exclusion criteria. The protocols of the current study were consistent with the ethical guidelines of the 2008 Helsinki Declaration. Institutional Review Board of Korea University Medical Center Anam Hospital ensured appropriate ethical and bioethical conduct and specifically approved this study. Written informed consent was waived since the current study is based on retrospective analysis.

### Ablation procedure

Ablation procedure in our institution can be summarized as follows. Quadripolar, decapolar, and duo-decapolar catheters were positioned at right ventricle or superior vena cava, high right atrium, and coronary sinus, respectively. Continuous blood pressure monitoring and blood sampling were conducted by left femoral artery line. Double trans-septal punctures were performed using Brockenbrough needle and two SL1 sheaths. After positioning of circular mapping and ablation catheters, 3 dimensional mapping of LA was performed with either EnSite NavX/Velocity (St. Jude Medical, St. Paul, Minnesota) or CARTO (Biosense Webster, Irvine, California) system. Wide antral pulmonary vein isolation was performed in all procedures. In patients with paroxysmal AF, trigger focus evaluation was performed by AF induction with rapid atrial pacing under isoproterenol infusion and subsequent direct current cardioversion. End point of the procedure in paroxysmal AF patients was the absence of trigger focus. If non-pulmonary vein trigger was present, additional ablation was performed to eliminate non-pulmonary vein trigger. In persistent AF, rapid atrial pacing was performed to induce AF without isoproterenol infusion after pulmonary vein isolation. If no sustained AF (lasting for more than 5 minutes) was induced, the procedure was finished. Additional ablation such as complex fractionated atrial electrogram guided ablation or linear ablation were performed on operator’s discretion if sustained AF was induced after pulmonary vein isolation. End point of the procedure was the absence of inducibility. Additional substrate modification was also permitted for paroxysmal AF if the operator considered substrate modification to be more important than trigger point elimination.

### Cardiac MRI

Follow up cardiac MRI was recommended during outpatient visit to all patients who had pre-RFCA MRI evaluation. Cardiac MRI was performed using a 3 T MR system (Achieva; Philips Medical Systems, Best, Netherlands) with a 32-element phased-array cardiac coil. Cardiac MRI data were acquired by using contrast-enhanced timing robust angiography sequence without ECG gating after injection of 0.2 mmol/kg of gadolinium contrast agent (Dotarem; Guerbet, S.A., Villepinte, France). The parameters of contrast-enhanced timing robust angiography sequence were as follows: parallel imaging using the sensitivity encoding technique with R = 2; acquisition time = 5 sec; a voxel size = 1 mm × 1 mm × 1.2 mm; TR/TE = 3.9 ms/1.1 ms; flip angle = 25°; bandwidth = 1106.2 Hz/pixel. Contrast media was injected at a rate of 2 ml/s via 18-gauge needle in the left or right arm vein by an automated power injector (Spectris Solaris EP; Medrad, Indianola, PA, USA); this was followed by an injection of a 40 ml normal saline bolus at the same rate. Using a commercially available software (Terarecon iNtuition; TeraRecon, Foster City, CA, USA), cardiac MRI data were evaluated by radiologist who specializes in the field of cardiac MRI (S.H. Hwang). The reviewer was blinded to all of the clinical data and measured the volume of LA and LAA. The LA and LAA cavities completely filled with gadolinium contrast agent were three-dimensionally reformatted. The reviewer traced and determined the endocardial boundaries of LA and LAA on cardiac MRI. The selected endocardial boundaries of LA and LAA excluded the pulmonary veins. Then, the LAA neck as the narrowest portion of LAA at the entrance into the LA was also selected to differentiate between the LA and LAA. Based on manually drawn endocardial boundaries of LA and LAA, the cardiac MRI voxels corresponding to the LA and LAA were selected respectively, and computed to measure the volumes (in mL) of LA and LAA. All measurements were re-evaluated and confirmed by Y.G. Kim and J. Shim and in case of any disagreement, it was resolved by discussion and re-tracing of endocardial boundaries. Representative images for LA and LAA volume measurements are presented in Supplementary Fig. S1.

### Transesophageal echocardiography (TEE)

All patients underwent TEE evaluation before RFCA to confirm the absence of thrombus in LA and LAA. Routine views (high esophageal 0°, 45°, 60°, and 120° views) were obtained to evaluate LA, left ventricle (LV), right chambers, and valves. LAA was evaluated using at least three different views (high esophageal 0°, 45°, 60°, or 120° views). Thorough evaluations of LA and LAA were performed to reveal any evidence of SEC or thrombus. SEC was classified into grade 1 (very mild; minimal echogenicity; only detectable transiently; increasing gain setting required for the detection), grade 2 (mild; detectable without increasing gain setting), grade 3 (moderate; dense and swirling echogenic material; echogenic signal is denser in LAA compared to LA), or grade 4 (severe; dense and swirling echogenic material; echogenic signal is equivocal in LAA and LA). Dense SEC was defined as a composite of moderate and severe SEC. During LAA imaging, emptying (forward) and filling (backward) flow velocity of LAA were measured using pulsed wave Doppler analysis. All TEE evaluations were accompanied by transthoracic echocardiography (TTE) evaluations.

### Outcome endpoints

The aim of the current study was to evaluate the impact of RFCA on LA and LAA volumes. Post-RFCA LA and LAA volumes measured with MRI were compared with pre-RFCA LA and LAA volumes respectively. LAA to LA volume ratio was defined as [LAA volume/(LA volume + LAA volume)]*100. The influence of clinical and procedural factors on ∆LA and ∆LAA volumes were also evaluated. The impact of AF type, presence of late recurrence, additional substrate modification, anterior line block with consequent delayed activation of LAA, and total ablation time were evaluated. The association between MRI findings and late recurrence, which was defined as any atrial tachyarrhythmia lasting for more than 30 seconds, was examined.

### Statistical analysis

Continuous variables are expressed as mean ± standard deviation (SD). Categorical variables are presented as percentile values. Unpaired t-test and one-way ANOVA test were used to compare continuous variables. Paired t-test was performed to compare pre-RFCA LA and LAA volumes with post-RFCA LA and LAA volumes respectively. Categorical variables were compared with chi-square test or Fisher’s exact test as appropriate. Pearson product-moment correlation analysis was performed to examine correlation between two continuous variables. Receiver operating characteristic curve analysis was performed to evaluate predictive value of continuous variables for any specific state variable. Comparison of area under curve (AUC) of two ROC curves was performed by statistical method suggested by Hanley and McNeil^26^. Subgroup analysis was performed to examine clinical and procedural factors associated with ∆LA and ∆LAA volumes. Cox regression analysis was performed to calculate hazard ratio (HR) of late recurrence for each variable. All significance tests were two-tailed and p values of less than 0.05 were considered statistically significant. All statistical analyses were performed with SPSS version 21.0 (IBM, Armonk, NY, USA).

### Data availability

All data generated or analysed during this study are included in this published article (and its Supplementary Information files).

## Results

### Patient characteristics

**Figure 1:**
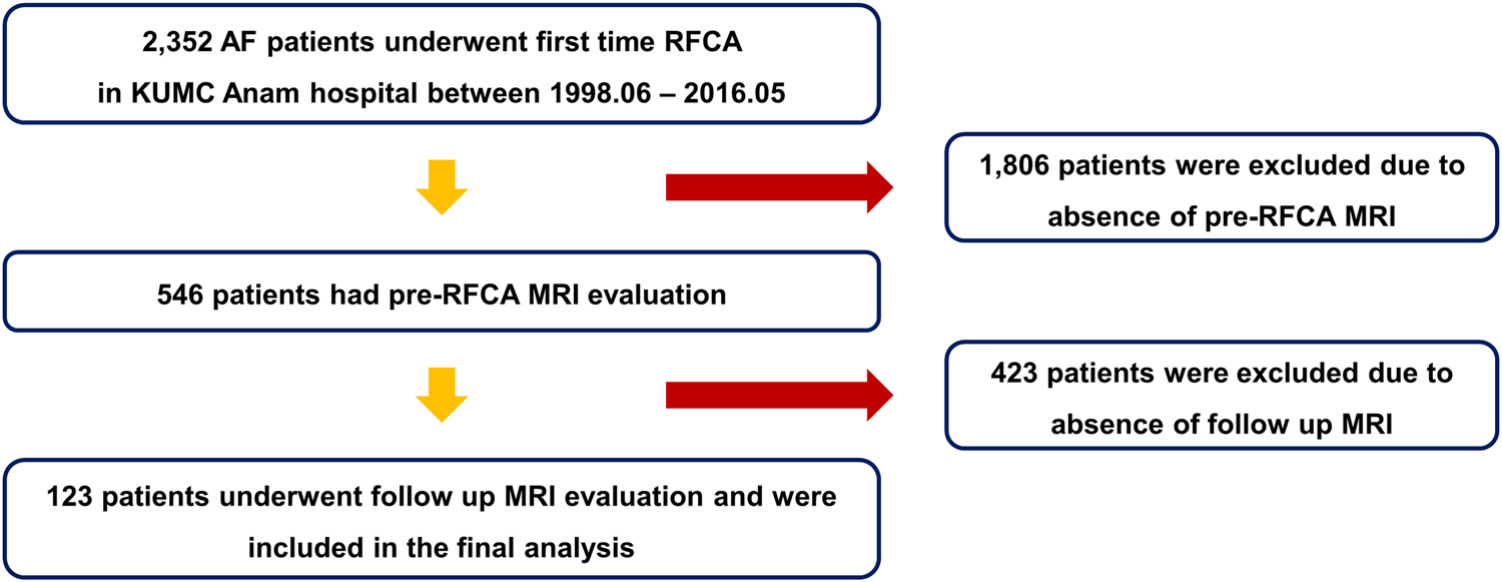
Flow diagram of the study.

Between June 1998 and May 2016, a total of 2,352 patients with AF underwent first-time RFCA in Korea University Anam hospital. Cardiac MRI was performed just before the procedure in 546 patients. Remaining 1,806 patients who underwent computed tomography evaluation or did not perform any imaging modalities before RFCA were excluded from the current study. Among 546 patients who performed cardiac MRI before RFCA, 312 patients who completed 1 year follow up after first-time RFCA were indicated for follow up MRI and 123 patients who actually underwent follow up MRI were included in the final analysis (Fig. 1).

Baseline characteristics of 123 patients are summarized in Table 1. Mean age was 55.1 ± 10.6 years old and 80.5% of patients were male. Fifty three (43.1%) patients were diagnosed with paroxysmal AF. Mean CHA_2_DS_2_-VASc score was 1.2 ± 1.3. During follow up, 78 (63.4%) patients experienced late recurrence.

**Table 1:** Baseline characteristics of the study patients.

	N = 123
Age (years)	55.1 ± 10.6
Male sex	99 (80.5%)
Paroxysmal	53 (43.1%)
Congestive heart failure	5 (4.1%)
Hypertension	42 (34.1%)
Diabetes mellitus	10 (8.1%)
Cerebrovascular events	12 (9.8%)
Vascular disease	4 (3.2%)
Baseline LA volume (ml)	101.2 ± 43.3
Baseline LAA volume (ml)	19.4 ± 8.5
Average LAA flow velocity (cm/sec)	41.3 ± 20.8
LV ejection fraction (%)	54.2 ± 6.0
Hemoglobin (g/dl)	14.9 ± 1.3
WBC (×10^3^/ul)	6.4 ± 1.5
Platelets (×10^3^/ul)	200.2 ± 39.6
INR	1.4 ± 0.6
Creatinine (mg/dl)	1.0 ± 0.2
CHA_2_DS_2_-VASc	1.2 ± 1.3
Follow up duration (days)	715.9 ± 227.8
Mean MRI follow up duration (days)	420.5 ± 171.5
Substrate modification	46 (37.4%)
Ablation time (min)	108.4 ± 56.6
Late recurrence	78 (63.4%)

INR: international normalized ratio; LA: left atrium; LAA: left atrial appendage; LV: left ventricle; MRI: magnetic resonance imaging; WBC: white blood cell count.

### Change in LA and LAA volume after RFCA

**Figure 2:**
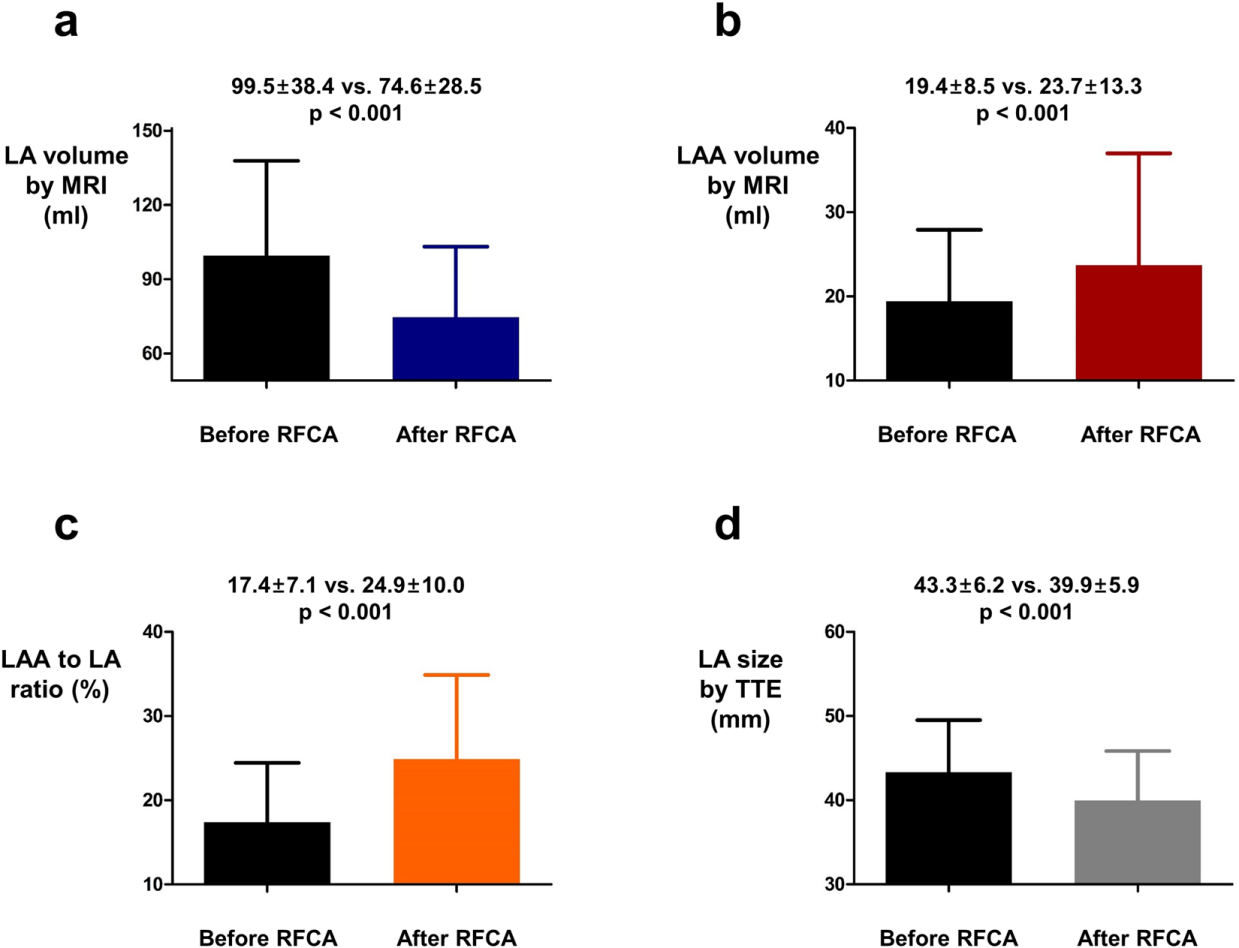
The influence of RFCA on LA and LAA volume. **(a)** LA volume was significantly decreased after RFCA. **(b**,**c)** LAA volume and LAA to LA volume ratio were significantly increased after RFCA. **(d)** LA diameter measured by TTE was also significantly decreased after RFCA. LA: left atrium; LAA: left atrial appendage; RFCA: radiofrequency catheter ablation; TTE: transthoracic echocardiography.

After undergoing RFCA, LA volume measured with MRI was significantly decreased (99.5 ± 38.4 ml vs. 74.6 ± 28.5 ml; p < 0.001; Fig. 2a). However, LAA volume (19.4 ± 8.5 ml vs. 23.7 ± 13.3 ml; p < 0.001; Fig. 2b) and LAA to LA volume ratio (17.4 ± 7.1% vs. 24.9 ± 10.0%; p < 0.001; Fig. 2c) measured with MRI were significantly increased following RFCA. LA diameter measured by TTE on parasternal long axis view (43.3 ± 6.2 mm vs. 39.9 ± 5.9 mm; p < 0.001; Fig. 2d) was significantly decreased after RFCA similar to LA volume measured with MRI. ∆LAA and ∆LA volumes of individual patients are depicted in Supplementary Figs S2 and S3 respectively.

### Impact of RFCA on LAA flow velocity and SEC

**Figure 3:**
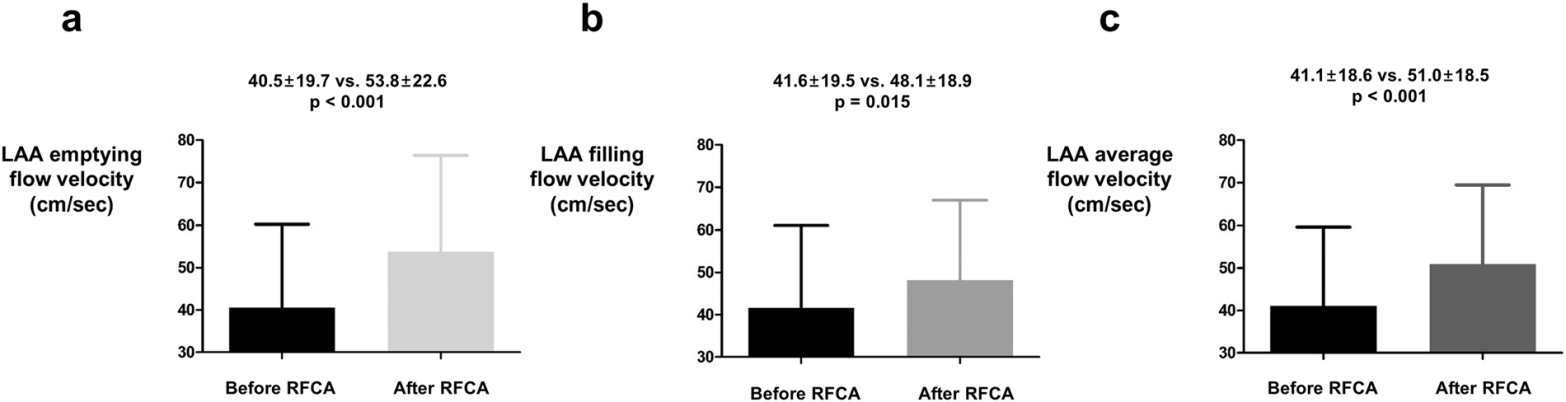
The influence of RFCA on LAA flow dynamics. Emptying **(a)**, filling **(b)**, and its average **(c)** flow velocities of LAA were significantly improved after RFCA. LA: left atrium; LAA: left atrial appendage; RFCA: radio frequency catheter ablation.

**Figure 4:**
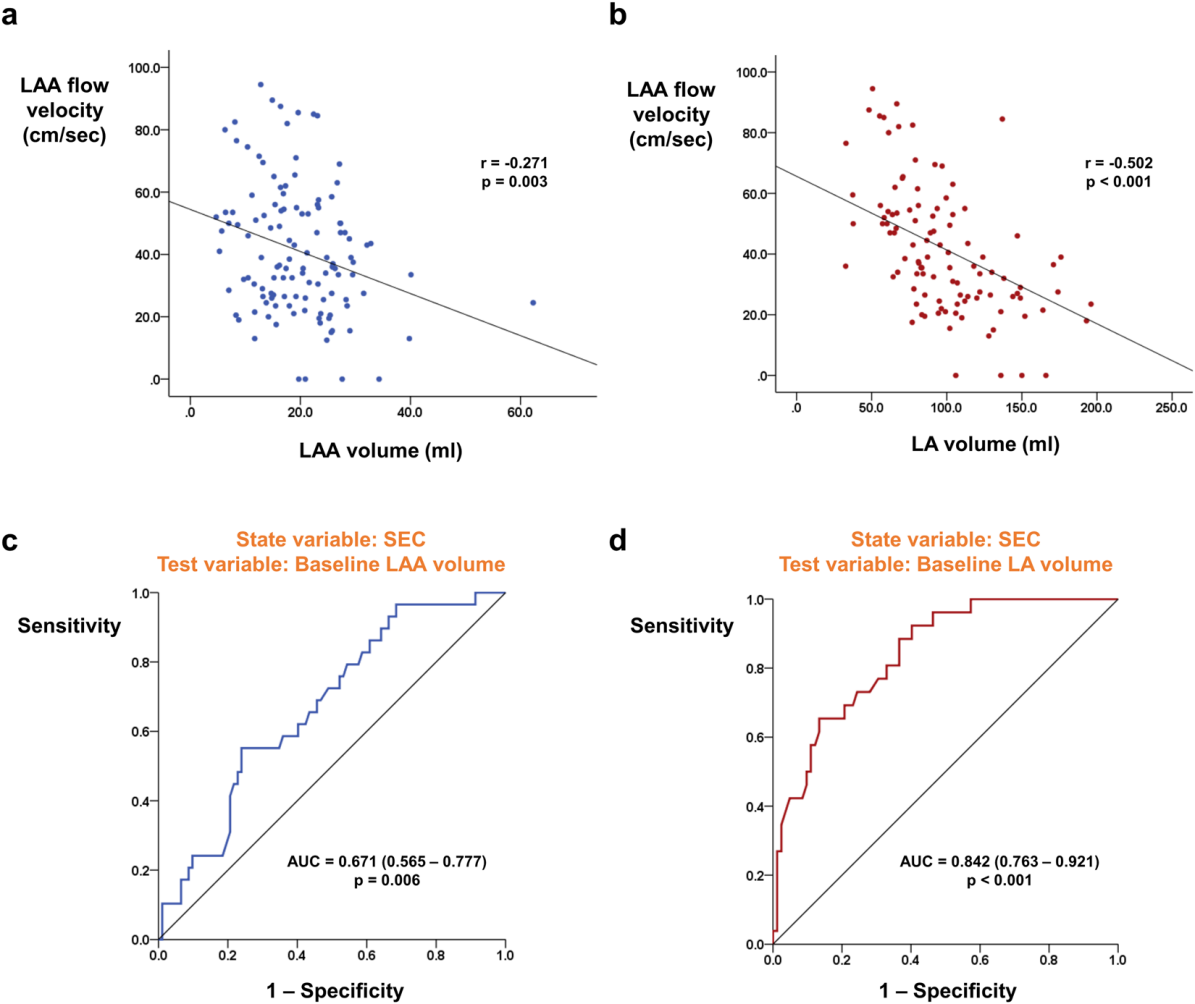
The association between baseline LA and LAA volume with TEE findings. LA volume showed more close association with both average LAA flow velocity **(a**,**b)** and presence of SEC **(c**,**d**) as compared with LAA volume. LA: left atrium; LAA: left atrial appendage; SEC: spontaneous echocontrast; TEE: transesophageal echocardiography.

**Figure 5:**
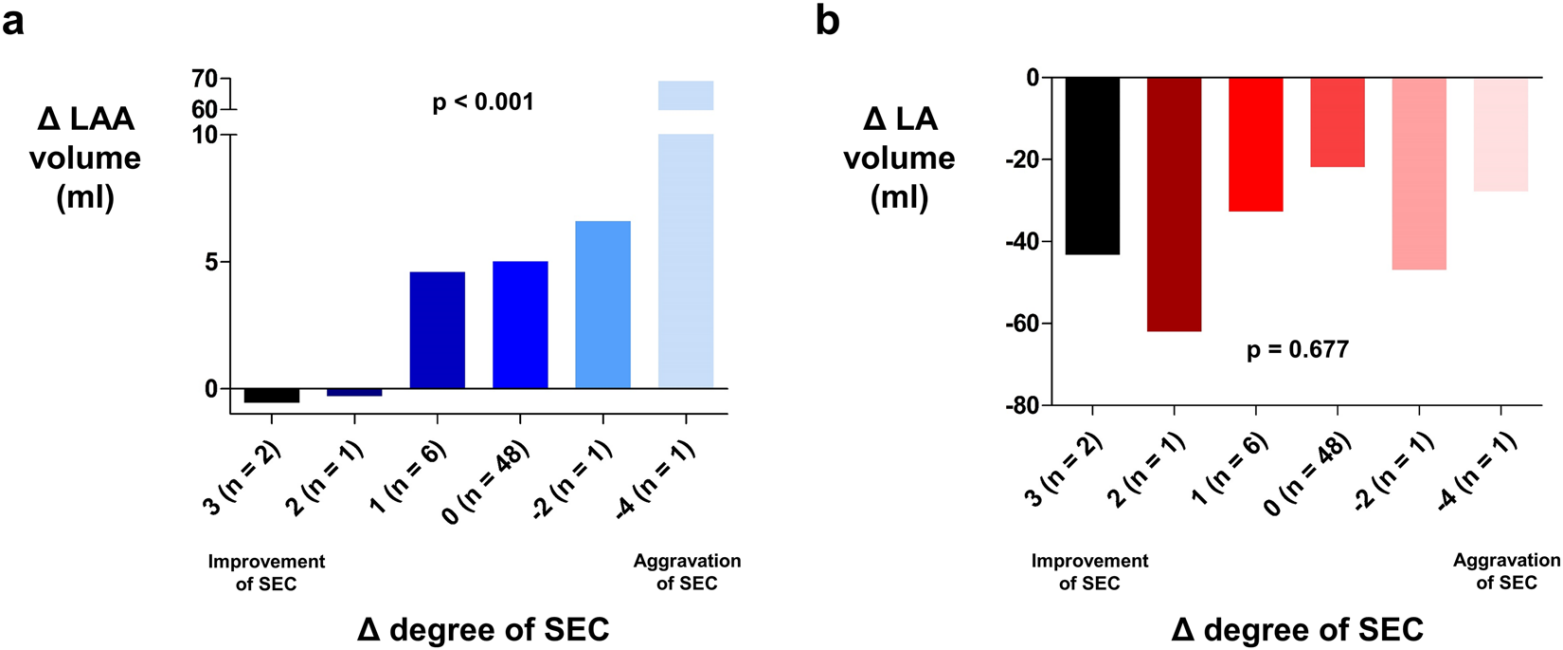
Relationship between change in SEC grade and ∆LA & ∆LAA volume. Significant difference in ∆LAA volume was present among different groups stratified change in SEC grade **(a)**. However, ∆LA volume showed no such association **(b)**. LA: left atrium; LAA: left atrial appendage; SEC: spontaneous echocontrast.

Among 123 patients included in the analysis, 59 patients had both baseline and follow up measurement of LAA flow velocity during TEE examination. The most frequent indication for follow up TEE study was re-do RFCA for recurred AF in 57 patients. Both forward (emptying; 40.5 ± 19.7 cm/sec vs. 53.8 ± 22.6 cm/sec; p < 0.001; Fig. 3a) and backward (filling; 41.6 ± 19.5 cm/sec vs. 48.1 ± 18.9 cm/sec; p = 0.015; Fig. 3b) flow velocity of LAA was significantly increased after RFCA. Average flow velocity of LAA was also improved after RFCA (41.1 ± 18.6 cm/sec vs. 51.0 ± 18.5 cm/sec; p < 0.001; Fig. 3c). Average ∆LAA flow velocity of individual patients are presented in Supplementary Fig. S4. Rhythm status during follow up TEE evaluation was recorded and among 59 patients, 34, 18, and 7 patients had sinus rhythm, atrial tachycardia, and AF respectively. Rhythm status had a significant impact on average LAA flow velocity with sinus rhythm and atrial tachycardia showing significantly higher LAA flow velocity as compared with AF (Supplementary Fig. S5). Sixty patients had both baseline and follow up TEE evaluation for SEC. Although baseline LAA volume showed significant correlation with LAA flow velocity, the degree of correlation was weaker than baseline LA volume (Fig. 4a,b). LA volume also outperformed LAA volume to predict the presence of SEC (AUC: 0.842 vs. 0.671; p < 0.001; Fig. 4c,d). However, ∆LAA volume showed better association with degree of change in SEC grade as compared with ∆LA volume. Nine patients showed improvements in SEC grade after undergoing RFCA. Two patients showed aggravation of SEC and 48 patients did not show any change in SEC grade following RFCA. Degree of improvement or aggravation in SEC grade showed association with ∆LAA volume but not with ∆LA volume (Fig. 5a,b).

### Impact of substrate modification on LA and LAA volume

**Figure 6:**
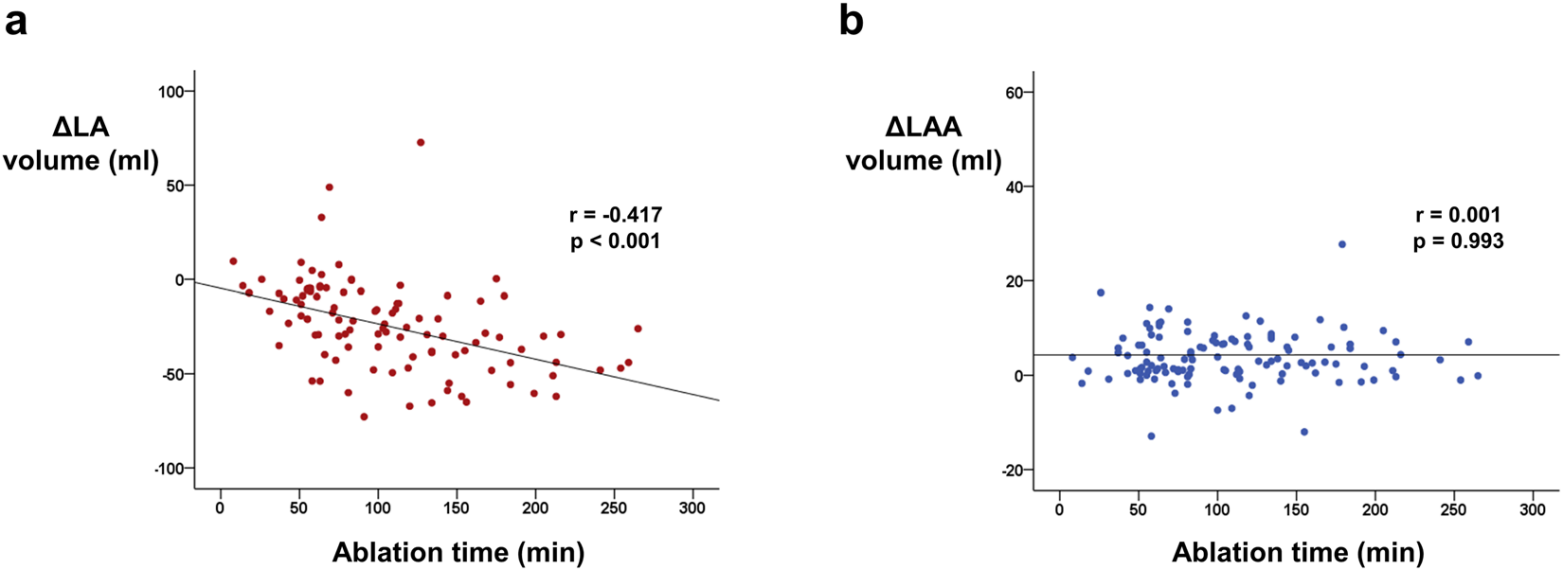
Impact of total ablation time on change in LA and LAA volume. Longer ablation time was associated with greater decrease in LA volume **(a)**. However, no association was found between total ablation time and ∆LAA volume **(b)**. LA: left atrium; LAA: left atrial appendage.

Patient with (n = 78) or without (n = 45) late recurrence showed no difference in ∆LA volume (−25.6 ± 29.2 ml vs. −23.7 ± 19.5 ml; p = 0.716; Table 2) and ∆LAA volume (5.0 ± 9.2 ml vs. 3.1 ± 5.4 ml; p = 0.218; Table 2). Non-paroxysmal AF (n = 70) as compared with paroxysmal AF (n = 53), showed larger baseline LA volume (114.9 ± 44.3 ml vs. 83.7 ± 35.3 ml; p < 0.001; Table 2) and greater decline in LA volume after RFCA (−29.6 ± 23.8 ml vs. −18.9 ± 27.5 ml; p = 0.034; Table 2). However, ∆LAA volume did not differ between the two groups (5.0 ± 9.6 ml vs. 3.4 ± 5.3 ml; p = 0.254; Table 2). Substrate modification in addition to pulmonary vein isolation (n = 77) was significantly associated with greater LA volume shrinkage after RFCA (−33.5 ± 26.3 ml vs. −12.1 ± 19.3 ml; p < 0.001; Table 2). In contrast, LAA volume was not affected by additional substrate modification (4.7 ± 9.2 ml vs. 3.6 ± 5.5 ml; p = 0.459; Table 2). Delayed activation of LAA results in simultaneous contraction of LAA and LV and therefore, might predispose LAA to higher intraluminal pressure. Successful block of anterior line (n = 31), confirmed by delayed activation of LAA, was associated with significant decrease in LA volume (−39.1 ± 28.1 ml vs. −20.8 ± 23.9 ml; p = 0.002; Table 2; see Supplementary Fig. S6 for representative intracardiac electrogram of delayed activation of LAA occurring simultaneously or after QRS complex). ∆LAA volume was numerically larger in anterior line block group (6.2 ± 12.8 ml vs. 3.7 ± 5.6 ml; p = 0.127; Table 2), but without statistical significance. Decrease in LA volume and increase in LAA volume after RFCA were consistent throughout all subgroups (Table 2). Total time for ablation was significantly associated with ∆LA volume with longer ablation time associated with greater shrinkage of LA (r = −0.417; p < 0.001; Fig. 6a), but not with ∆LAA volume (r = 0.001; p = 0.993; Fig. 6b).

**Table 2:** Impact of clinical parameters on LA and LAA size.

	No late recurrence (n = 45)			Late recurrence (n = 78)			p value (between group)
	Mean	SD	p value (pre and post)	Mean	SD	p value (pre and post)	
Baseline LA	93.4	31.7		105.7	48.4		0.153
F/U LA	69.6	25.2		77.0	29.6		0.166
∆ LA	−23.7	19.5	<0.001	−25.6	29.2	<0.001	0.716
Baseline LAA	20.4	6.7		18.9	9.3		0.336
F/U LAA	23.6	8.4		23.8	15.4		0.921
∆ LAA	3.1	5.4	<0.001	5.0	9.2	<0.001	0.218
	**Paroxysmal (n = 53)**			**Non-paroxysmal (n = 70)**			
Baseline LA	83.7	35.3		114.9	44.3		<0.001
F/U LA	66.0	23.1		80.4	30.2		0.004
∆ LA	−18.9	27.5	<0.001	−29.6	23.8	<0.001	0.034
Baseline LAA	19.3	6.3		19.6	9.9		0.834
F/U LAA	22.6	7.7		24.5	16.4		0.433
∆ LAA	3.4	5.3	<0.001	5.0	9.6	<0.001	0.254
	**PVI only (n = 46)**			**PVI + Substrate modification (n = 77)**			
Baseline LA	77.8	23.6		117.0	46.5		<0.001
F/U LA	65.0	24.5		79.7	28.9		0.005
∆ LA	−12.1	19.3	<0.001	−33.5	26.3	<0.001	<0.001
Baseline LAA	20.2	6.5		19.0	9.5		0.420
F/U LAA	23.8	8.3		23.7	15.6		0.957
∆ LAA	3.6	5.5	<0.001	4.7	9.2	<0.001	0.459
	**No anterior line block (n = 92)**			**Anterior line block (n = 31)**			
Baseline LA	97.6	42.3		113.7	45.2		0.108
F/U LA	74.7	28.2		73.0	28.6		0.780
∆ LA	−20.8	23.9	<0.001	−39.1	28.1	<0.001	0.002
Baseline LAA	19.9	7.3		18.0	11.3		0.380
F/U LAA	23.5	8.5		24.2	22.4		0.873
∆ LAA	3.7	5.6	<0.001	6.2	12.8	0.012	0.127

F/U: follow up; LA: left atrium; LAA: left atrial appendage; PVI: pulmonary vein isolation; SD: standard deviation.

### MRI findings and risk of late recurrence

Both baseline and follow up MRI findings were not associated with the risk of late recurrence. In Cox regression analysis, HR was 1.002 (p = 0.421), 1.006 (p = 0.182), and 1.002 (p = 0.712) for baseline LA, follow up LA, and ∆LA volume respectively. LAA volume also was not associated with late recurrence: HR = 0.979 (p = 0.121) for baseline LAA; HR = 0.996 (p = 0.605) for follow up LAA, and HR = 1.007 (p = 0.528) for ∆LAA.

## Discussion

The findings of the current study can be summarized as follows: (i) RFCA is associated with decreased LA volume (ii) However, LAA volume is increased after RFCA (iii) additional substrate modification and total ablation time are associated with greater decrease in LA volume (iv) LAA volume was increased after RFCA irrespective of presence of late recurrence, type of AF, procedure type, and ablation time (v) flow velocity of LAA is increased even in patients with late recurrence. Previous studies focused on the impact of RFCA on LA but not on LAA. However, it is LAA which have more profound impact on ischemic stroke in AF patients and therefore, influence of RFCA on LAA deserves more attention. The current study evaluated LA and LAA simultaneously using MRI and TEE in AF patients undergoing RFCA.

### Decreased LA volume and increased LAA volume after RFCA

Previous studies consistently reported decreased size (or volume) of LA after RFCA in AF patients^27^. However, in contrast to LA size or volume, LA ejection fraction is not improved after RFCA^25,27^. In the study performed by John and his colleagues, LA end-systolic volume was decreased after RFCA but LA ejection fraction was also reduced^25^. Furthermore, reduced LA volume and systolic function was closely associated with the volume of LA scar related to RFCA^25^. Therefore, LA volume reduction after RFCA might not be a result of improved hemodynamics in LA but rather due to LA scar formation and shrinkage of LA which is often called ‘stiff LA syndrome’^28^. Our results support this concept. Patients who underwent additional substrate modification and longer duration of radiofrequency energy delivery showed greater decline in LA volume as compared with patients with pulmonary vein isolation only. Furthermore, the amount of LA and LAA volume change did not differ between patients with successful ablation and those who experienced late recurrence indicating that the change in LA and LAA volume is not the result of successful restoration of sinus rhythm but consequences of scar created by radiofrequency energy.

Increased LAA volume after RFCA also deserves further attention. LAA volume, area, and orifice area are known risk factors for ischemic stroke in patients with AF^17,18^. The current study demonstrated that LAA volume is increased after RFCA in AF patients. Furthermore, enlargement of LAA volume was independent from the presence of late recurrence, AF type, procedure type, and ablation time. The mechanism of increased LAA volume after RFCA is uncertain. Ablation scar might result in stiff LA wall which will not dilate sufficiently to receive incoming blood from pulmonary veins. As a consequence, LA pressure will rise and relatively compliant LAA might dilate in response to increased pressure in LA which is similar to a balloon effect. After RFCA, ∆LA volume did not show any association with ∆degree of SEC but ∆LAA volume revealed a significant association with ∆degree of SEC with increased LAA volume correlated with aggravation of SEC. Whether this increased LAA volume will actually lead to ischemic stroke in AF patients undergoing RFCA needs further investigation. Previous studies demonstrated that low LA compliance, represented by elevated LA pressure and decreased LA pulse pressure, is associated with electroanatomical remodeling of the LA and increased risk of late recurrence after AF ablation^29,30^. Whether RFCA, especially extensive ablation including LA substrate modification, actually leads to elevated LA pressure is not directly demonstrated in our study. However, increase in LAA volume in contrast to decreased LA volume raise a concern about adverse hemodynamic effect of RFCA on LA and LAA. Future studies should measure pre- and post-RFCA LA pressure to better understand the effect of RFCA on LA hemodynamics.

### Increased flow velocity of LAA after RFCA

The mechanism of ischemic stroke in AF patients is still not conclusive. However, it is generally accepted that blood stasis caused by AF, especially in LAA, makes a suitable place for the development of fibrin-rich red thrombus^14,31,32^. The underlying pathophysiology of thrombus formation is significantly different from acute coronary syndrome in which platelets play a major role. Since the activation of coagulation cascade triggered by flow stasis is one of the main mechanism of thrombus formation and consequent ischemic stroke in AF patients, anticoagulants show significantly better efficacy compared to antiplatelets in terms of stroke prevention^33,34^. LAA, especially when blood stasis is accompanied, is the major source of thrombus formation and consequent cardio-embolic stroke. Therefore, it is not surprising that the flow velocity of LAA measured with TEE is a significant predictor of future ischemic stroke in AF patients^16^. Maintaining adequate emptying and filling velocity of LAA will have a significant benefit in terms of stroke prevention. According to our analysis, emptying, filling, and its average flow velocity of LAA were improved after RFCA. The results are in contrast to our previous analysis regarding LAA volume since RFCA was associated with increased LAA volume. The reason for this discrepancy is not clear, but we assume that LAA flow velocity was improved after RFCA since substantial number of patients were in atrial tachycardia, rather than atrial fibrillation, at the time of late recurrence (43 and 35 patient recurred as AF and AT respectively). The clinical importance of increased LAA flow velocity associated with RFCA is currently not determined and needs further evaluation.

### Limitations

The current study have several limitations. First, this was a retrospective study. Our analyses are not free from intrinsic limitations of retrospective analysis. Second, follow up TEE evaluation was usually performed in patients with late recurrence. Follow up MRI was also preferentially performed in patients who experienced late recurrence.

## Conclusions

In conclusion, LA volume is decreased after RFCA which is probably due to stiff LA related to scar formation. LAA volume, in contrast to LA, is increased after RFCA. Clinical significance of increased LAA volume after RFCA needs further evaluation in future trials.

## Electronic supplementary material

Supplementary Figures
