# Supplementary material for: Different Responses of Left Atrium and Left Atrial Appendage to Radiofrequency Catheter Ablation of Atrial Fibrillation: a Follow Up MRI study
Source: Sci Rep. 2018 May 18;8:7871. doi: 10.1038/s41598-018-26212-y (PMC5959897; doi:10.1038/s41598-018-26212-y)
Supplement: Supplementary file 1 — Supplementary Figures [file 41598_2018_26212_MOESM1_ESM.pdf]

Different Responses of Left Atrium and Left Atrial Appendage to Radiofrequency Catheter Ablation of Atrial Fibrillation: a Follow Up MRI study

Yun Gi Kim,[1] Jaemin Shim,[1]* Suk-Kyu Oh,[1] Hee-Soon Park,[1] Kwang-No Lee,[1] Sung Ho Hwang,[2] Jong-Il Choi,[1] and Young-Hoon Kim[1]

[1] Division of Cardiology, Department of Internal Medicine, Korea University Medical Center Anam Hospital, Seoul, Republic of Korea

[2] Department of Radiology, Korea University Medical Center Anam Hospital, Seoul, Republic of Korea

* **Correspondence**

Jaemin Shim, MD, PhD

Division of Cardiology, Department of Internal Medicine, Korea University College of Medicine and Korea University Medical Center

73, Inchon-ro, Seongbuk-gu, Seoul 02841, Republic of Korea

Tel: 82-2-920-5445

Fax: 82-2-927-1478

E-mail: jaemins@korea.ac.kr

Running title: LAA enlargement after RFCA

Supplementary Figure legends

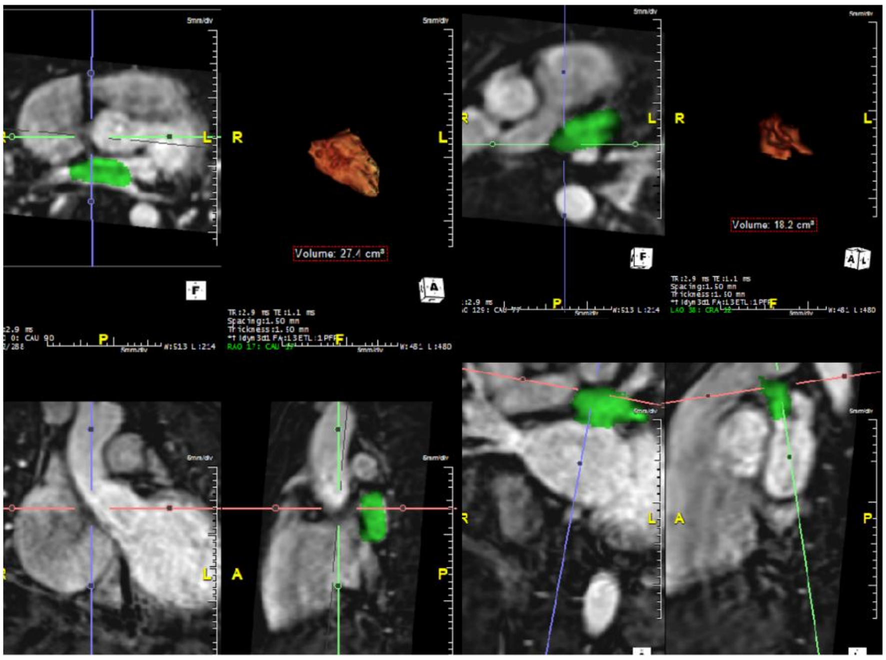

Supplementary Figure S1. Representative images for demonstration of LA and LAA volume measurement based on cardiac MRI.

LA: left atrium; LAA: left atrial appendage, MRI: magnetic resonance imaging.

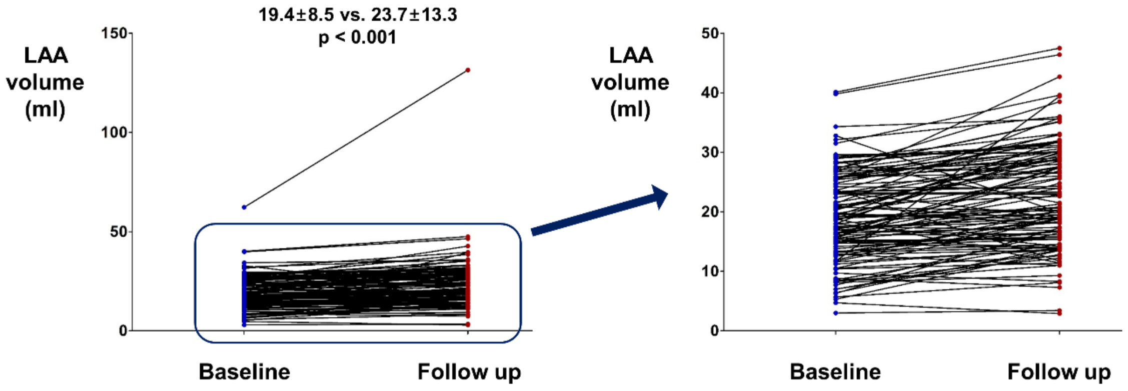

Supplementary Figure S2. ΔLAA volume of individual patients.

LAA volume was significantly increased after RFCA.

LAA: left atrial appendage; RFCA: radiofrequency catheter ablation.

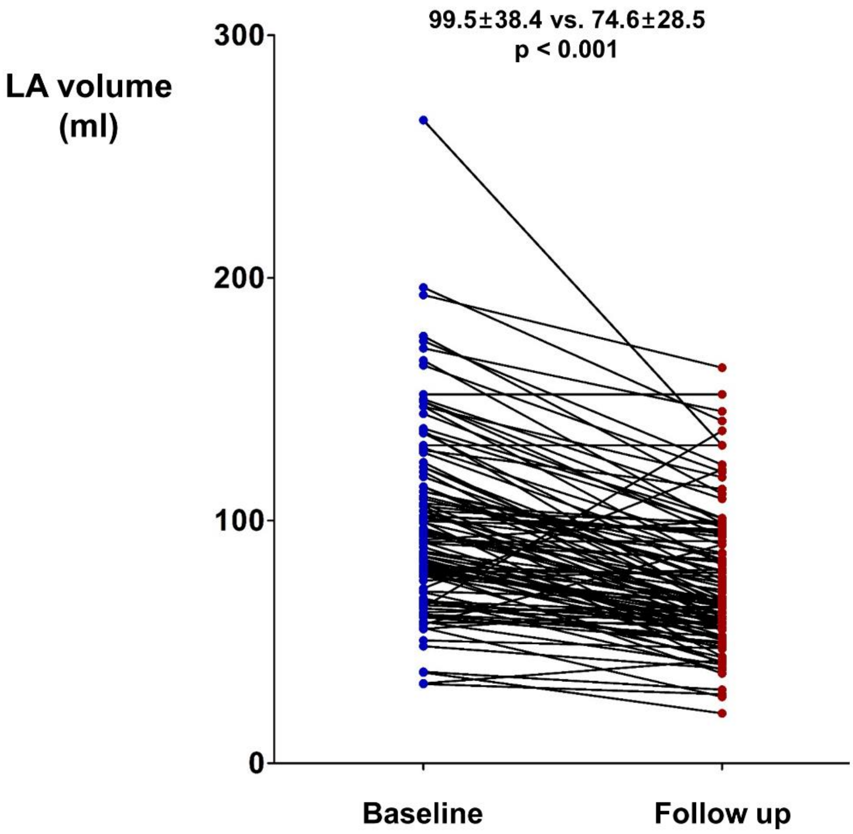

Supplementary Figure S3. ∆LA volume of individual patients.

After RFCA, substantial proportion of patients showed decreased LA volume.

LA: left atrium; RFCA: radiofrequency catheter ablation.

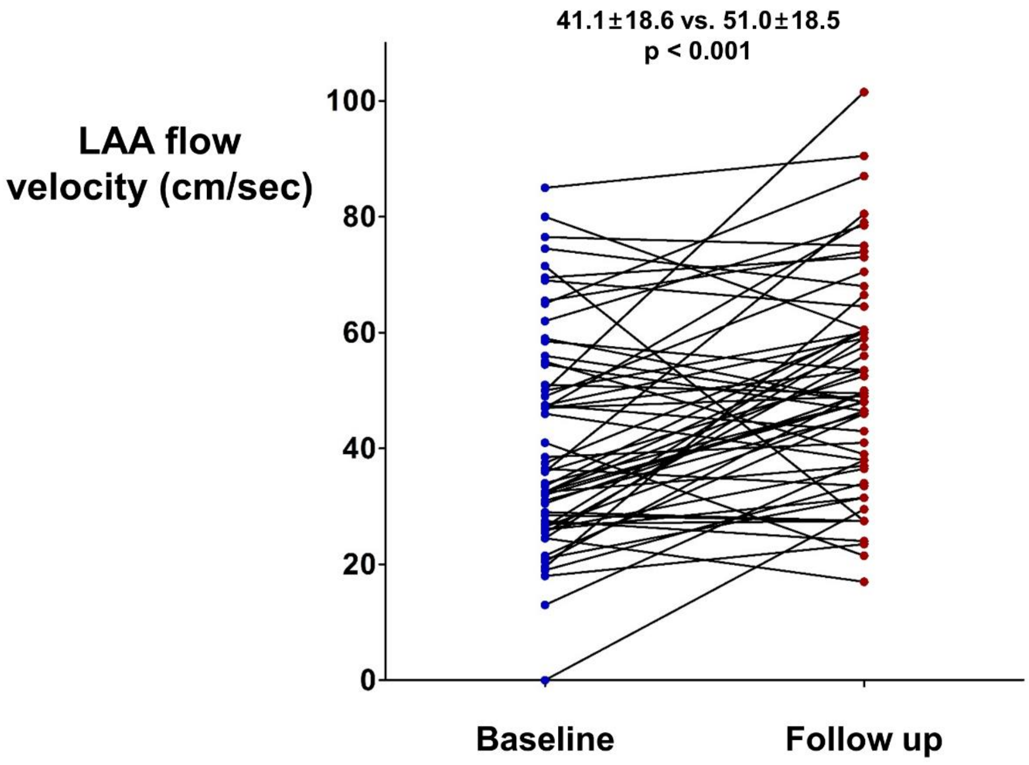

Supplementary Figure S4. ΔLAA average flow velocity of individual patients.

Average LAA flow velocity was significantly increased in follow up (post-RFCA) TEE evaluation.

LAA: left atrial appendage; RFCA: radiofrequency catheter ablation; TEE: transesophageal echocardiography.

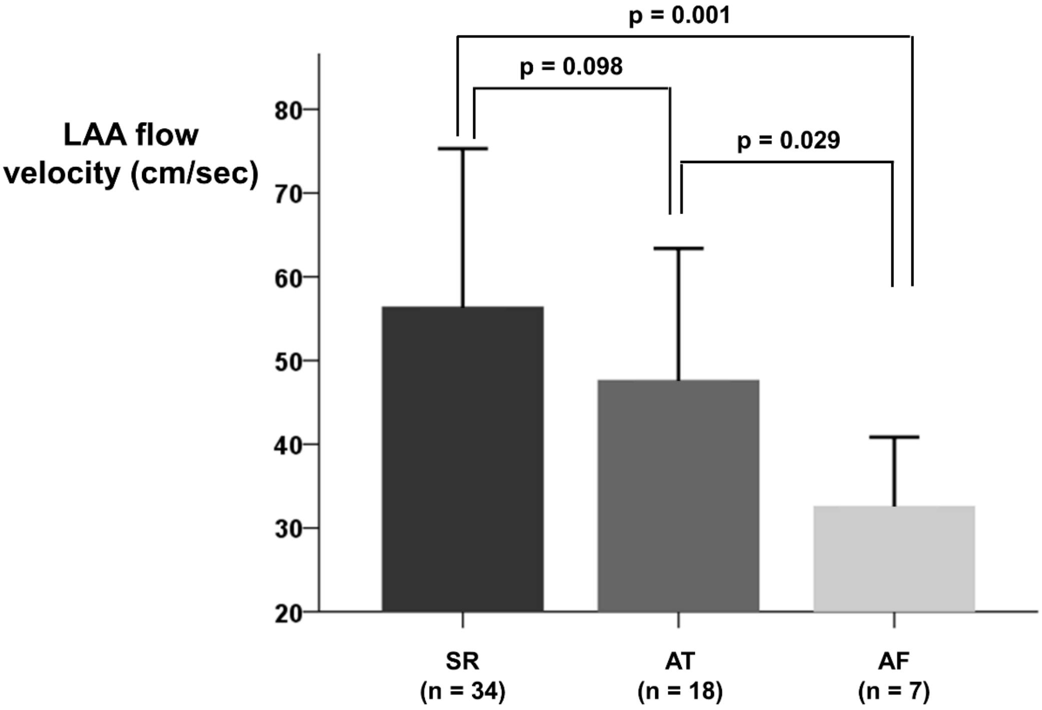

Supplementary Figure S5. Average LAA flow velocity during follow up TEE according to rhythm status.

During follow up TEE evaluation, average LAA flow velocity significantly differed among patients with SR, AT, and AF.

AF: atrial fibrillation; AT: atrial tachycardia; LAA: left atrial appendage; SR: sinus rhythm; TEE: transesophageal echocardiography.

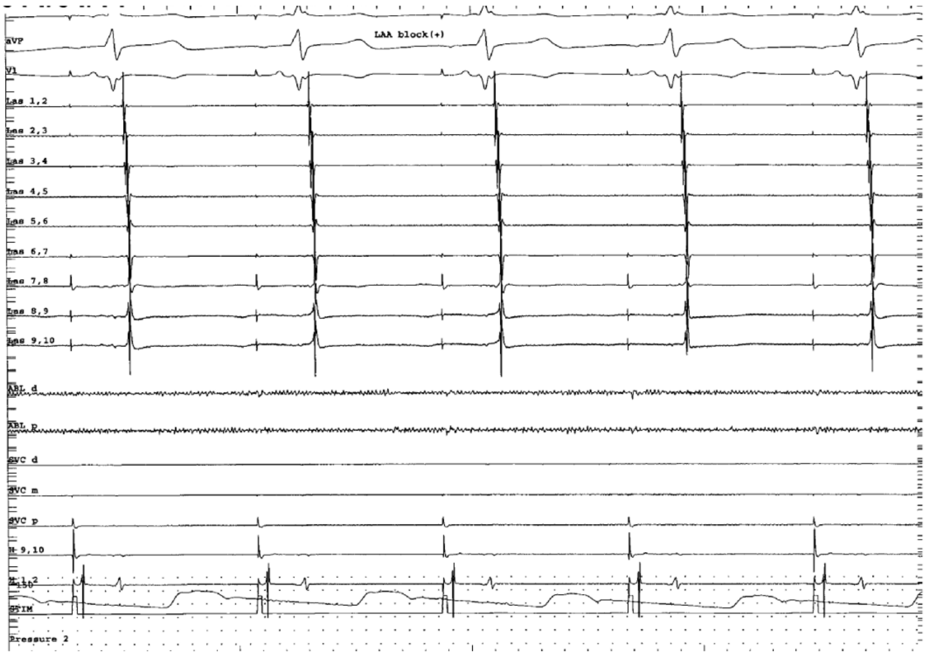

Supplementary Figure S6. Representative electrogram of delayed activation of LAA.

Anterior line block resulted in delayed activation of LAA. In this case, activation of LAA was even slower than ventricles. Circular mapping catheter was placed in LAA. Left atrium was paced with right atrial catheter.

LAA: left atrial appendage.
